# An Ensemble Approach to Knowledge-Based Intensity-Modulated Radiation Therapy Planning

**DOI:** 10.3389/fonc.2018.00057

**Published:** 2018-03-19

**Authors:** Jiahan Zhang, Q. Jackie Wu, Tianyi Xie, Yang Sheng, Fang-Fang Yin, Yaorong Ge

**Affiliations:** ^1^Department of Radiation Oncology, Duke University Medical Center, Durham, NC, United States; ^2^Department of Software and Information Systems, University of North Carolina at Charlotte, Charlotte, NC, United States

**Keywords:** treatment planning, dose volume histogram prediction, regression model, machine learning, ensemble model, statistical modeling

## Abstract

Knowledge-based planning (KBP) utilizes experienced planners’ knowledge embedded in prior plans to estimate optimal achievable dose volume histogram (DVH) of new cases. In the regression-based KBP framework, previously planned patients’ anatomical features and DVHs are extracted, and prior knowledge is summarized as the regression coefficients that transform features to organ-at-risk DVH predictions. In our study, we find that in different settings, different regression methods work better. To improve the robustness of KBP models, we propose an ensemble method that combines the strengths of various linear regression models, including stepwise, lasso, elastic net, and ridge regression. In the ensemble approach, we first obtain individual model prediction metadata using in-training-set leave-one-out cross validation. A constrained optimization is subsequently performed to decide individual model weights. The metadata is also used to filter out impactful training set outliers. We evaluate our method on a fresh set of retrospectively retrieved anonymized prostate intensity-modulated radiation therapy (IMRT) cases and head and neck IMRT cases. The proposed approach is more robust against small training set size, wrongly labeled cases, and dosimetric inferior plans, compared with other individual models. In summary, we believe the improved robustness makes the proposed method more suitable for clinical settings than individual models.

## Introduction

In radiation therapy, high quality treatment plans are crucial for reducing the possibility of normal tissue complications while maintaining good dose coverage of planning target volume (PTV). For intensity-modulated radiation therapy (IMRT), it is especially important to fully utilize the healthy tissue sparing potential enabled by the advanced treatment delivering system. However, the optimal achievable organ-at-risk (OAR) sparing is not known pre-planning, and planners need to rely on their previous experience, which makes the planning process subjective, iterative, and susceptible to intra- and inter-planner variation.

Knowledge-based planning (KBP) (1–5) has been shown to be a powerful tool for guiding planners and physicians to optimal achievable OAR dose volume histograms (DVHs) based on previous cases planned by experienced planners. In a previously proposed regression-based KBP framework (2), the workflow is as follows: (i) principle component analysis (PCA) is conducted for OAR DVHs in the training set, and the first three principle component scores (PCS) and corresponding basis vectors are stored; (ii) pre-determined geometry information related to treatment planning goals, also referred to as features, are calculated for each patient; (iii) PCS of OAR DVH are fitted to features to generate a prediction model; (iv) features are calculated for new patients; and (v) best achievable OAR DVHs are calculated for new patients using the fitted model and the previously calculated PCA basis vectors.

In step (iii) of the previous framework, stepwise regression is used to select features and estimate the linear model. The method automatically picks several most important features step by step based on the significance of features. This approach is easy to implement and the output is interpretable. With careful training data preprocessing and feature selection, stepwise has achieved good results in OAR DVH prediction in research settings (6–12). However, there are some theoretical issues about this procedure, which could potentially result in some instabilities of the overall model training process. While stepwise regression has been very successful in the context of KBP, potential disadvantages of stepwise regression are well documented. First, it potentially suffers from overfitting if the size of the training set is relatively small compared to the number of features. This is because the procedure attempts to fit many models and the *p*-values, which are used as feature selection criteria, are not corrected for the number of hypothesis tested. In addition, stepwise regression does not cope with collinear features well. If two features are highly collinear, stepwise usually selects just one and discard the other. Ideally, if several collinear features are predictive of the outcome, all of these features should be selected to prevent overfitting and reduce model variance.

The purpose of this study is to improve the regression modeling aspect of KBP. Empirically, different regression methods perform well in different scenarios, such as different number of training cases, presence of collinear features, and presence of outlier cases. In this work, we develop an ensemble learning method to combine the strengths of these individual models and improve KBP model robustness.

## Materials and Methods

### Individual Models

As a comparison to our proposed ensemble model, we study four individual regression models, including ridge regression (13, 14), lasso (15), elastic net (16), and stepwise regression with forward feature selection. These models also serve as base learners for the final ensemble model. The latter three models share the same objective function

(1) $$\beta =\text{argmin}\left\{{\left\|Y-X\beta \right\|}_{2}^{2}+\varphi \left(\beta \right)\right\},$$

where $X\in {\mathbb{R}}^{N\times P}$ denotes *P* feature value from *N* training cases, $Y\in {\mathbb{R}}^{N}$ denotes OAR DVH PCS of cases in the training set, and $\beta \in {\mathbb{R}}^{P}$ denotes regression coefficients corresponding to *P* anatomical features, such as PCS of distance-to-target histogram. Detailed descriptions of feature extraction and dimension reduction for KBP can be found in Ref. (1, 2). The last term, known as the penalty term, balances the bias and variance of the trained model. The goal of KBP is to obtain regression coefficients β based on cases previously planned by experienced planners, and when a new case needs to be planned, the optimal OAR DVH can be calculated simply using the model predicted PCS of *X*β. In ridge regression, the penalty term φ(β) is the square of ℓ2-norm of the regression coefficients β; in lasso, the penalty term is the ℓ1-norm of β; and in elastic net, the penalty term is simply a linear combination of ℓ1-norm and ℓ2-norm squared:

(2) $$\varphi \left(\beta \right)=\lambda_{1}{\left\|\beta \right\|}_{1}+\lambda_{2}{\left\|\beta \right\|}_{2}^{2}.$$

The penalty weights λ_1_ and λ_1_ are selected based on internal cross validation.

Forward selection, a type of stepwise regression, is the last individual model. It finds the most significant features to add based on the data step by step, hence the name. When adding features no longer improves the model by a certain preset *p*-value threshold, the feature selection step terminates. The selected features are fitted to the data with ordinary least square, while the rest of the features are discarded.

### The Ensemble Model

Many ensemble models have been proposed over the years in the field of machine learning, such as random forest (17), boosting (18), bagging (19), and stacking (20). The basic idea behind these ensemble models is to develop an array of simple models, often referred to as base learners, and combine these models to form a better (e.g., lower variance, higher accuracy, or both) model for prediction (21). These models essentially seek to combine knowledge learned by different models *via* data resampling and/or adding another layer of optimization.

The primary motivation of our ensemble model is to make KBP more robust and adaptive. In different settings, different regression models perform well, and none of these individual models consistently performs better than other models. For instance, stepwise regression is widely known to be unstable (22), but as shown in Section “Results,” it can significantly outperform other more stable models such as ridge regression in certain settings. However, it is not feasible to test out individual models every time a new model is fit. Therefore, we propose an ensemble model, which performs well in all settings.

#### Model Stacking

In our proposed model, we combine the aforementioned individual models using model stacking method. A previous study demonstrated that even stacking ridge regression alone with different penalty weight λ improved model generalization performance, and stacking models with different characteristics generated further improvement (20). The proposed ensemble approach is shown in Eqs 3–5

(3) $$z_{\mathrm{kn}}=\beta_{k}x_{n},k=1,K,$$

(4) $$\alpha_{k}^{\text{*}}=\underset{\alpha_{k}}{\text{argmin}}\sum_{n=1}^{N}{\left(y_{n}-\sum_{k=1}^{K}\alpha_{k}z_{\mathrm{kn}}\right)}^{2},\text{ s.t. }\forall \alpha_{k}\geq 0,$$

(5) $$Y=\sum_{k=1}^{K}\alpha_{k}^{\text{*}}\beta_{k}X\text{.}$$

First, individual models β*_k_*, where *k* ∈ [1, *K*] denotes individual model index, are trained separately on the training dataset repetitively with all the training data except for case *n*. Prediction of the in-training-set but out-of-model case *z_kn_* is then generated (Eq. 3). The process is repeated until all the models have covered all cases in the training set. Subsequently, the model weights $\alpha_{k}^{\text{*}}$ are optimized to minimize internal cross validation error, as shown in Eq. 4. A non-negative constraint is applied to prevent overfitting and increase the model interpretability. This step of optimization is done on the metadata, and the prediction results of each model for each case are used to optimize the model weights. The individual models that perform well in the prediction task tend to get larger weightings. The *K* individual models β*_k_* are combined and used for prediction of DVH PCS *Y* (Eq. 5). Note that the sum of optimal model weights $\alpha_{k}^{\text{*}}$ is not constrained to 1, as one would intuitively expect. This is due to the distinct properties of the individual models in the ensemble. The regression coefficients by stepwise regression are usually too large due to lack of constraint and thus need shrinkage. On the contrary, the other three regression methods tend to under-fit, especially for noisy training data, i.e., data with high variance that cannot be explained by any features in *X*. In other words, even if we have just one model in the “ensemble,” the model weight is still highly unlikely to be 1 (usually smaller than 1 for stepwise and greater than 1 for penalized linear regression methods). In practice, we observe the sum of $\alpha_{k}^{\text{*}}$ is usually between 0.5 and 1.5.

**Figure 1:**
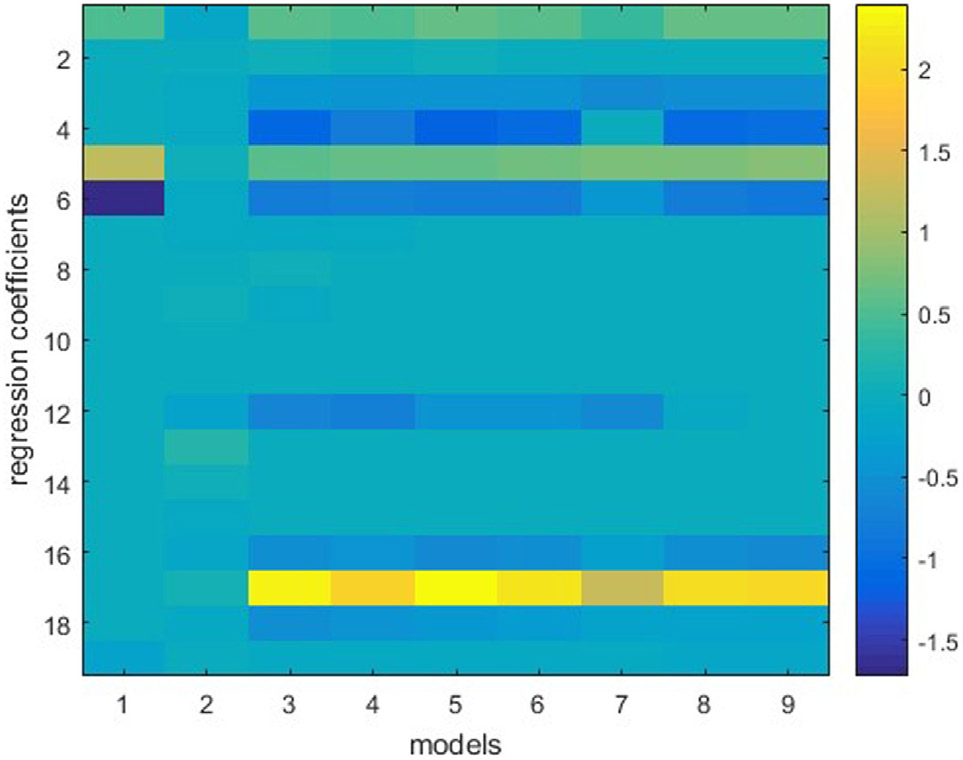
Individual models trained on the same dataset. Vertical lines represent regression coefficients of individual models. Models 1–9 (from left to right) refer to stepwise regression, ridge regression, six elastic net regression models with various parameters, and lasso. The vertical bar on the right indicates color mapping. Note that stepwise regression uses the least (four) features and ridge regression uses all features but assigns small weights to the features.

The ensemble in this study consists of nine models, including stepwise, ridge, lasso, and elastic net with six different λ_2_-to-λ_1_ ratios. Figure 1 shows one example of the model weights from the individual models. This model is built using 50 prostate sequential boost cases. *Y* is the bladder DVH PCS1, and *X* consists of bladder anatomical features. All features are standardized before training, thus the weights of different features are in the same scale. It is apparent that regression coefficients differ from model to model, even though these are all variants of linear regression models. Note that model 1, stepwise regression, uses the least number of features, and model 2, ridge regression, evidently underfits.

### Model-Based Case Filtering

In previous studies, it has been pointed out that automatic outlier removal requires further investigation (12, 23). We propose to incorporate a model-based automatic outlier removal routine in the ensemble model to ensure model robustness and address the volatile nature of clinical data. We utilize the cross validation metadata native to the proposed ensemble method to identify and remove impactful dosimetric and anatomical outliers. The two scenarios of outliers have different impact on the training of regression models, as we illustrate in this section. Note that by our definition outliers only exist in training sets, all cases in testing sets are predicted. Cases that would be defined as outlier cases if they are in a training set can still be predicted by a trained model, but with less accuracy. These special cases can be identified with the same approach as we identify outlier cases (see Model-Based Case Filtering Method), and case-based reasoning can be used to improve the outcome of treatment planning, but that is out of the scope of this study. We aim to improve prediction accuracy of the KBP framework with a different modeling technique, without significant changes to the overall workflow.

#### Outliers

Clinical treatment planning varies from case to case, with different sparing and coverage considerations. With the aforementioned KBP framework, we assume a linear model can successfully represent a majority of training cases. For some cases in the database, this assumption does not hold. We refer to these cases in the training dataset as outlier cases. In this section, we shall present our insight on outlier cases and provide an intuitive explanation of effects of outliers on knowledge-based modeling.

##### Anatomical Outliers and Dosimetric Outliers

**Figure 2:**
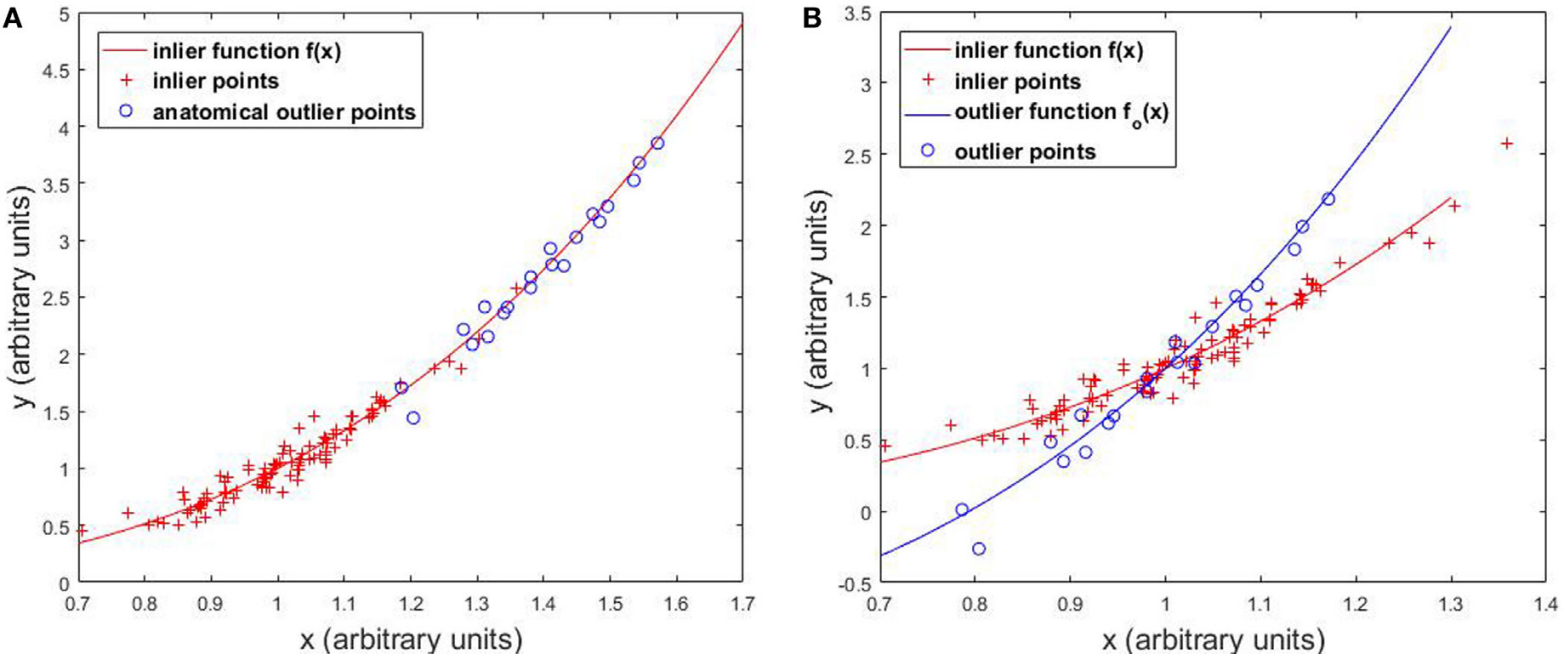
Effects of **(A)** anatomical outliers and **(B)** dosimetric outliers on the regression model.

The first type of outliers is anatomical outliers. In this study, we define anatomical outliers as cases with anatomical features that are distant from normal cases, and possibly come from a different distribution. In KBP, anatomical outliers refer to cases with uncommon anatomical features relevant to DVH prediction, such as abnormal OAR sizes, unusual OAR volume distributions relative to PTV surface. Generally, anatomical outliers are more likely to deviate from the linear model, as illustrated in Figure 2, and when they do, the effect of these cases are generally larger than normal cases due to the quadratic data fidelity term (first term in Eq. 1) of the regression model. Therefore, it is necessary to identify anatomical outlier cases that are detrimental to model building and remove those from the model before training.

Other than anatomical outliers, there are cases that are detrimental to model building due to limited OAR sparing efforts and/or capabilities. These are considered to be dosimetric outliers in this work. Dosimetric outliers include, but are not limited to (1) treatment plans with inferior OAR sparing and (2) wrongly labeled data, such as 3D plans mixed in IMRT plans.

##### Outliers’ Effect on Regression Models

In this section, we illustrate the effect of outliers on the overall regression model with one-dimensional simulated data. Figure 2A shows that anatomical outliers follow the same underlying *X*-to-*Y* mapping. However, the true underlying relation may not be well approximated by linear regression outside the normal *X* range. Attempting to fit linear regression with anatomical outliers mixed in the training set will potentially deteriorate the model. Therefore, the actual effect of anatomical outlier in different feature directions in the context of KBP needs careful assessment. Figure 2B illustrates the effect of dosimetric outliers. Dosimetric outliers in the training set are expected to increase model variance and deviate the model.

Note that this numerical demonstration isolates the effect of outliers on regression on a single feature, and it simplifies the influence of outliers on the overall modeling process. In our clinical knowledge-based modeling, we extract nine features from each case to construct the feature vector *X*. However, not every feature contributes to the final model equally. In stepwise regression, relevant features are picked based on correlation with the outcomes variable (i.e., DVH PCS). In penalized regression methods, features are implicitly selected with less relevant features given very small regression coefficients as a result of the penalty term. The feature selection step, while not considered here, is also affected by outliers. When anatomical outliers are involved in the training process, the features selected are potentially different from the set of features selected, if the model is trained without outliers.

#### Prediction Performance Measure

Weighted root mean squared error (wRMSE) is defined to evaluate model prediction accuracy:

(6) $$\text{wRMSE}=\sum_{i=1}^{N}w_{i}^{\prime }\;{\left({\text{DVH}}_{i}-\hat{{\text{DVH}}_{i}}\right)}^{2}.$$

Weighted root mean squared error measures the overall deviation of predicted DVHs from ground truth DVHs, which are clinically planned. Weightings are introduced to emphasize higher dose regions of DVHs, which are generally considered to be of more clinical significance in OAR dose predictions. Here $w_{i}^{\prime }=Nw_{i}/\sum_{j=1}^{N}w_{j}$ denotes the normalized weighting factor for bin *i* of DVH curves. For evaluation of dose to bladder and rectum, we use the linear relative weighting *w_j_* of 50–100 linearly increases from 0 Gy to prescription dose. For evaluation of dose to parotids in head and neck cases, *w_i_* is set to Gaussian centered at median dose, with SD of 2 Gy. If *w_i_* is set to a constant number, then wRMSE reduces to standard RMSE.

#### Model-Based Case Filtering Method

**Figure 3:**
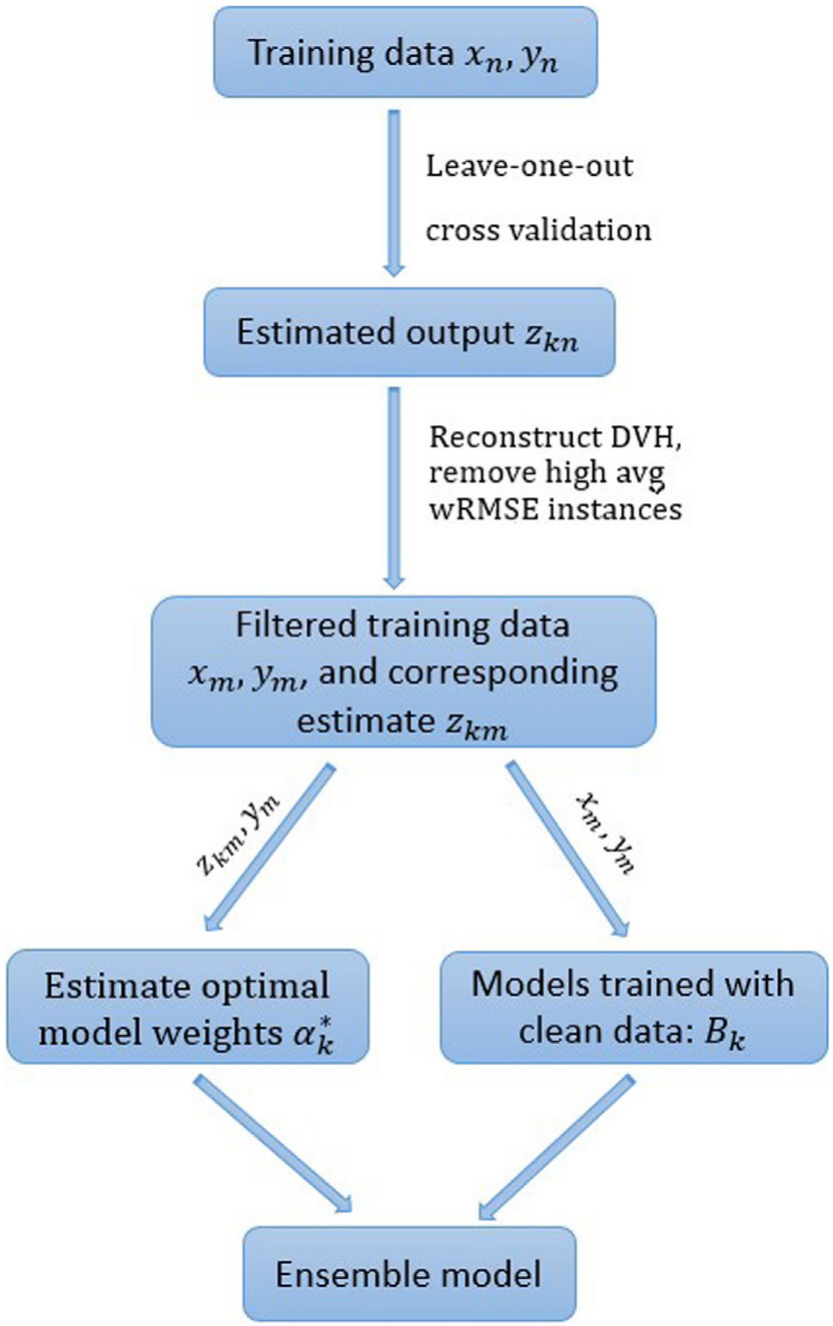
The proposed ensemble learning workflow.

To further improve the robustness of the ensemble model, cases with the highest *s*% median (of all individual models) internal cross validation wRMSE error are dropped from the training set. The percentage threshold *s* is selected to balance the tradeoff between model robustness and accuracy. Empirically, we find that 10% is generally a good choice, even though the number of actual outlier cases is unknown and may differ from 10% of the total case number. All the experiments in the following section are conducted with the pre-determined 10% threshold. The workflow of the ensemble model with model-based case filtering is shown in Figure 3. Note that the whole process is done automatically without manual intervention.

### Experimental Design

This retrospective study uses anonymized clinical plan data and has received permission from Duke University Medical Center’s institutional IRB. All clinical plans were planned using Varian Eclipse™ Treatment Planning System (Varian Medical Systems, Inc., Palo Alto, CA, USA). All experiments were performed on a PC with Intel Xeon E5-2623 CPU and 32 GB of RAM running Windows 10 Enterprise 64-bit operating system.

In order to quantitatively evaluate the robustness of these regression methods in various challenging clinical environment, we test the aforementioned models with limited training set size, training sets contaminated with anatomical outliers, and training sets contaminated with dosimetric outliers. In our outlier robustness tests, we purposefully mix pre-defined outlier cases into the training set and validate the final model with normal cases. The reason for adding outlier cases is to add controlled variation to the dataset and evaluate the robustness of the proposed model. Details regarding types of data used in the experiments are summarized in Table 1.

**Table 1:** Summary of data used in the experiments.

Experiments	Training data	Validation data
Limited training set size	20 prostate intensity-modulated radiation therapy (IMRT) cases	146 prostate IMRT cases

Anatomical outliers	10 prostate cases treated with lymph nodes and 40 prostate cases treated without lymph node	111 prostate cases treated without lymph node

Dosimetric outliers (inferior plans)	40 prostate IMRT cases and 10 prostate conformal arc plans	110 prostate IMRT plans

Dosimetric outliers (mis-classified sparing decisions)	80 bilateral parotid-sparing head and neck plans and 10 single-side sparing plans	148 bilateral parotid-sparing head and neck plans

#### Robustness to Limited Training Set Size

In clinical practice, planners do not necessarily have many cases for every treatment site. This is particularly true when a new treatment technique, such as simultaneous intensity boost, is recently utilized in the clinic and the existing model built for existing treatment techniques may not predict the achievable DVH accurately due to the OAR sparing capability difference. Sometimes models need to be built when only a small number of cases (~20) are available. It is critical that the regression model is capable of resisting overfitting the random variation of training cases. In this experiment, 166 prostate PTV cases are retrospectively retrieved from the clinical database. Twenty prostate cases are used as the training set, and the remaining 146 cases are used as validation set to quantitatively evaluate the prediction accuracy of each model.

#### Robustness to Anatomical Outliers

In clinical databases, not every previously treated case is helpful for predicting future cases even when the treatment plans are of high quality. If the anatomical features are very different from the majority of all cases than the linear assumption may not hold, as demonstrated in Figure 2, and the anatomical features are potentially detrimental to the model. To simulate the effect of anatomical outliers on the plans, we train a model with 10 prostate cases treated with lymph nodes and 40 prostate cases treated without lymph node. The trained models are subsequently validated with 111 cases that do not involve lymph nodes.

#### Robustness to Dosimetric Outliers

Dosimetric outliers do not follow the same conditional distribution as normal cases and are expected to be easier to be identified with cross validation. Increase of dosimetric outliers in training data tends to shift the overall model toward inferior plan DVHs and gradually make the plan less optimal (23). In this section, we evaluate the robustness of individual models and the ensemble model with training set contaminated by two types of dosimetric outlier plans: (i) inferior dose sparing and (ii) mis-labeled sparing decisions.

For KBP, it is crucial to get reliable predictions even in the presence of sub-optimal plans. Here, we simulate the sub-optimal plans with dynamic conformal arc plans. Compared with IMRT plans, conformal arc plans have evidently inferior OAR sparing capability. Our training data consists of 40 prostate IMRT cases and 10 prostate conformal arc plans, and the validation set includes 110 prostate IMRT plans. The experiment is designed to test the model robustness in the extreme settings to evaluate the model robustness in challenging situations.

In clinical practice, it is not always feasible to spare both parotids due to geometric factors. A previous study has shown that parotid-sparing decisions affect KBP predictions, and separate models should be built for single-side parotid sparing and bilateral parotid sparing to get better prediction accuracy (24). We retrieve 228 bilateral parotid-sparing head and neck cases and 10 single-side parotid-sparing cases from our institutional clinical database. The sparing decisions are first obtained from clinical prescription documentations and subsequently checked in dose statistics to correct for decision changes. We randomly select 80 bilateral cases as the training set and then add 10 single-side sparing cases as mis-classified cases. The remaining 148 bilateral cases are used as the validation set.

## Results

### Robustness to Limited Training Set Size

**Figure 4:**
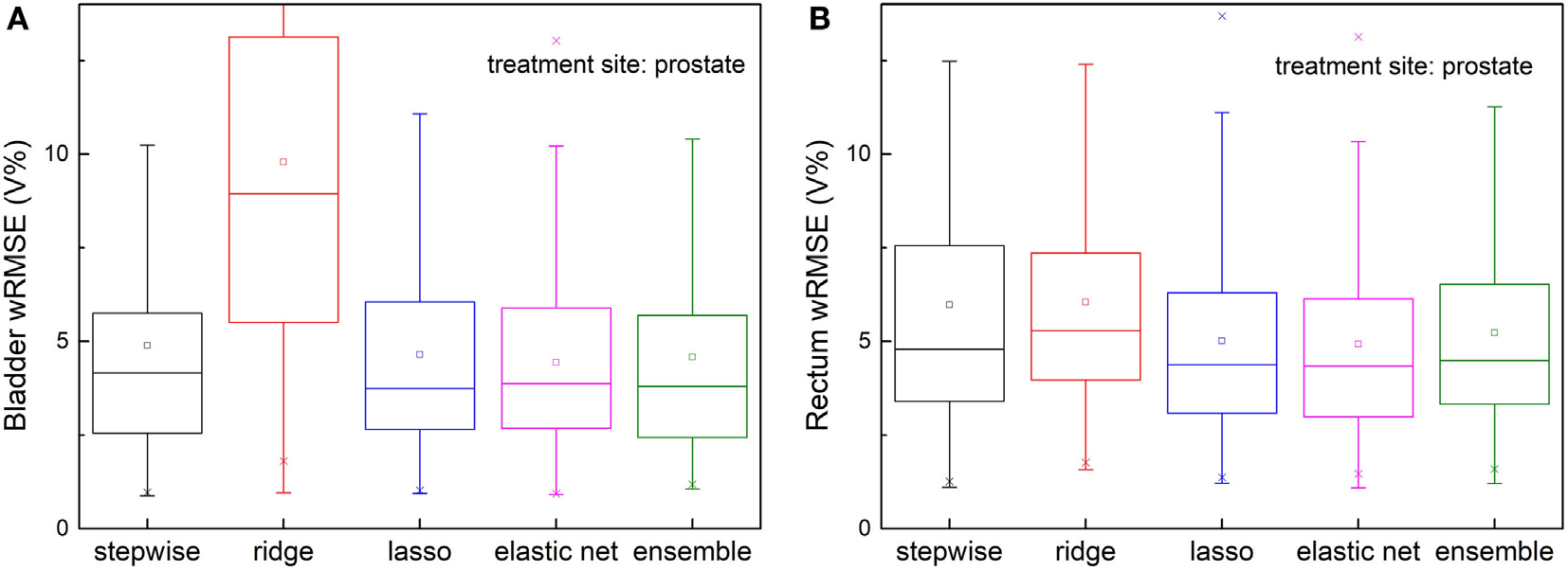
Prediction errors [weighted root mean squared error (wRMSE)] of individual regression models and the proposed ensemble model for **(A)** bladder **(B)** rectum. The training set and validation set for all the models tested are identical. Twenty prostate 1PTV cases are included in training set, and the validation set includes 146 cases. For bladder prediction, the proposed ensemble method predicts significantly better than stepwise (*p* < 0.001), ridge (*p* < 0.001), lasso (*p* < 0.001), and elastic net (*p* < 0.001); for rectum prediction, the proposed ensemble method predicts significantly better than ridge (*p* < 0.001), lasso (*p* < 0.001), elastic net (*p* < 0.001), and stepwise (*p* < 0.001).

The ensemble method outperforms all individual methods significantly, as shown in Figure 4. Note that ridge regression performs particularly poorly in bladder prediction, indicating that there is some intrinsic sparsity in the feature space, and ridge regression, which does not utilize that sparsity, underfits significantly due to over-shrinking of regression coefficients. Stepwise performs poorly in rectum predictions, due to overfitting.

### Robustness to Anatomical Outliers

**Figure 5:**
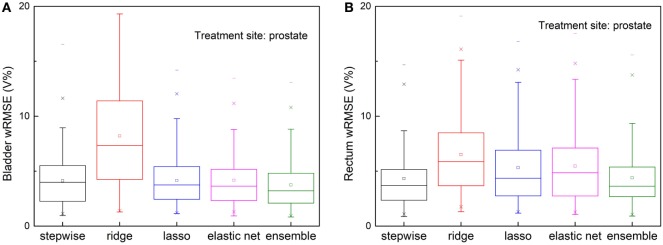
Prediction errors [Weighted root mean squared error (wRMSE)] of individual regression models and the proposed ensemble model for **(A)** bladder **(B)** rectum, in the presence of simulated anatomical outliers (see text). Forty prostate with seminal vesicle cases and 10 prostate cases with lymph node cases are used as the training set; 111 cases prostate with seminal vesicle cases are used as the validation set. For bladder prediction, the proposed ensemble method predicts significantly better than stepwise (*p* = 0.013), ridge (*p* < 0.001), lasso (*p* = 0.002), and elastic net (*p* < 0.001); for rectum prediction, the proposed ensemble method predicts significantly better than ridge (*p* < 0.001), lasso (*p* < 0.001), elastic net (*p* < 0.001), and performs similarly well as stepwise (*p* = 0.210).

Figure 5 shows prediction errors, measured by wRMSE, of individual models and the ensemble model. For bladder predictions, the ensemble model outperforms all individual models, while stepwise, lasso, and elastic net perform similarly. In the case of rectum predictions, the ensemble method again outperforms ridge, lasso, and elastic net, and performs similarly well as stepwise. Ridge regression fails to predict accurately for either task.

### Robustness to Dosimetric Outliers

#### Inferior Plans

**Figure 6:**
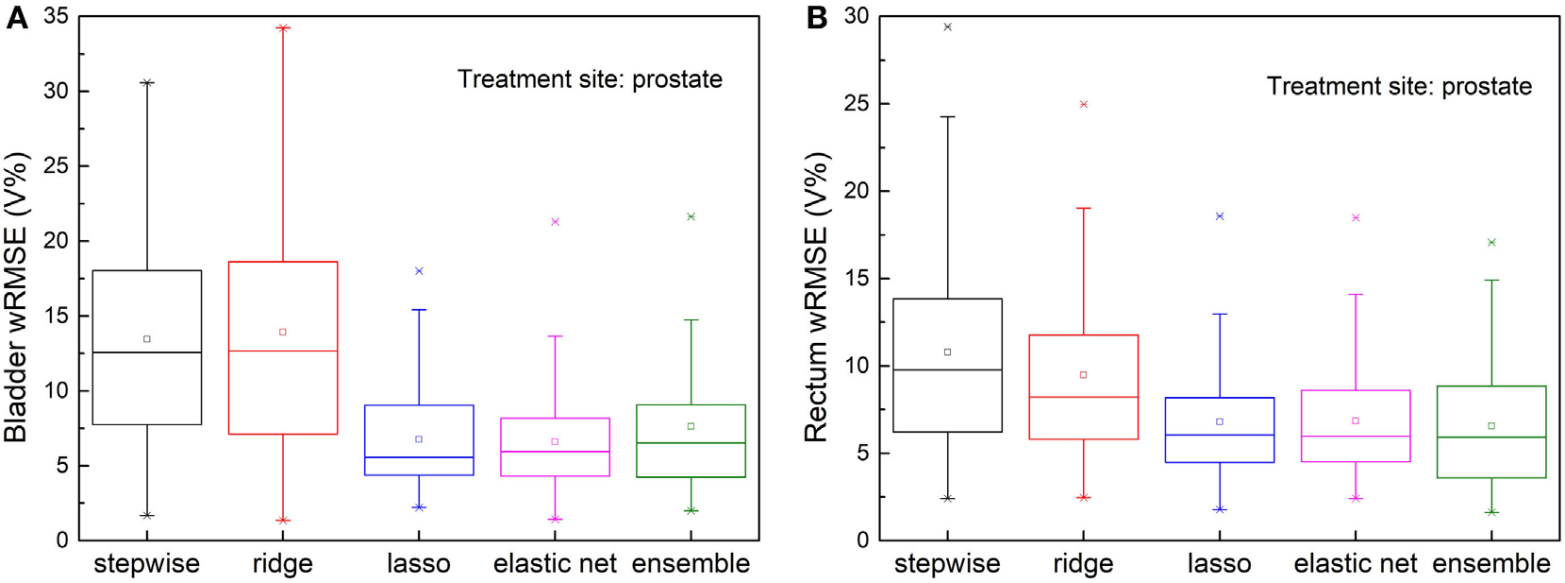
Prediction errors [Weighted root mean squared error (wRMSE)] of individual regression models and the proposed ensemble model for **(A)** bladder **(B)** rectum, in the presence of simulated dosimetric outliers (see text). For bladder prediction, the proposed ensemble method predicts significantly better than stepwise (*p* < 0.001), ridge (*p* < 0.001), and performs similarly well as lasso (*p* = 0.753) and elastic net (*p* = 0.841). For rectum prediction, the proposed ensemble method predicts significantly better than stepwise (*p* < 0.001) and ridge (*p* < 0.001), and performs similarly well as lasso (*p* = 0.365) and elastic net (*p* = 0.373).

Figure 6 shows, for both bladder and rectum prediction, lasso, elastic net, and the proposed ensemble regression method predict equally well, while stepwise and ridge are no longer usable due to significant amount of error.

#### Mis-Classified Sparing Decisions

**Figure 7:**
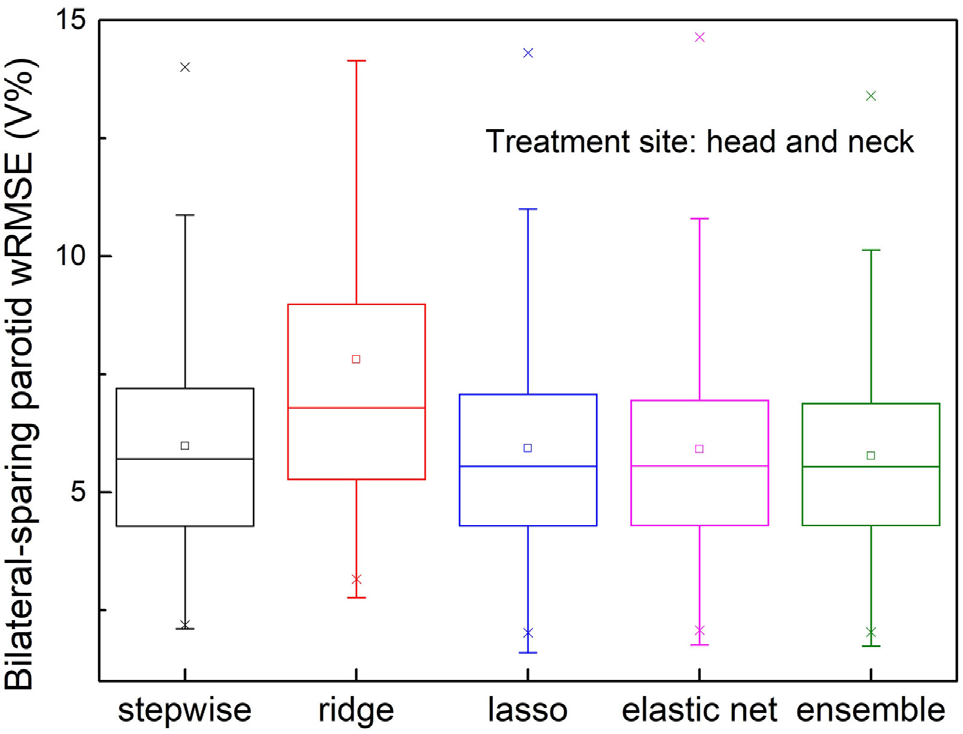
Prediction errors [Weighted root mean squared error (wRMSE)] of individual regression models and the proposed ensemble models for parotid dose volume histogram (DVH) prediction of bilateral parotid-sparing cases. Training set includes 80 bilateral sparing cases and 10 single-side sparing cases. Validation set includes 148 bilateral sparing cases. The proposed ensemble model yields significantly reduced prediction error than stepwise (*p* = 0.026) and ridge (*p* < 0.001), but does not outperform elastic net (*p* = 0.090) or lasso (*p* = 0.115).

The validation set prediction errors of each model are shown in Figure 7. The proposed ensemble model significantly reduces prediction error, compared with stepwise (*p* = 0.026) and ridge (*p* < 0.001), and performs equally well as elastic net (*p* = 0.091) and lasso (*p* = 0.115).

## Discussion

In summary, we propose an ensemble regression model to address two problems that we are facing in KBP. First, different individual regression models perform well in different settings, such as different number of relevant features, number of cases, and existence of outliers. It would be very labor intensive to manually select the optimal model every time a model is fitted. Second, to ensure the most accurate model training, data-preprocessing, including anatomical and dosimetric outlier removal, is also necessary for individual models, and it can be subjective to decide which subset of cases should be removed from the training set if done manually. The proposed ensemble model utilizes multiple individual models on the same set of data and uses constrained linear optimization on the metadata to obtain the optimal weight for each individual model. In addition, the model automatically filters out cases in the training set that are not predicative of future cases based on metadata.

We observe that the ensemble method consistently predicts better than or similar to the best performing individual model in every challenging situation. With improved robustness, the proposed regression method potentially enables end users to build site-specific, physician-specific, or even planner specific models, without manually screening the training cases. This eventually will allow each practice to build models that accurately reflect their own optimal OAR sparing preference and capability, thereby eliminating the need for a universal model.

**Figure 8:**
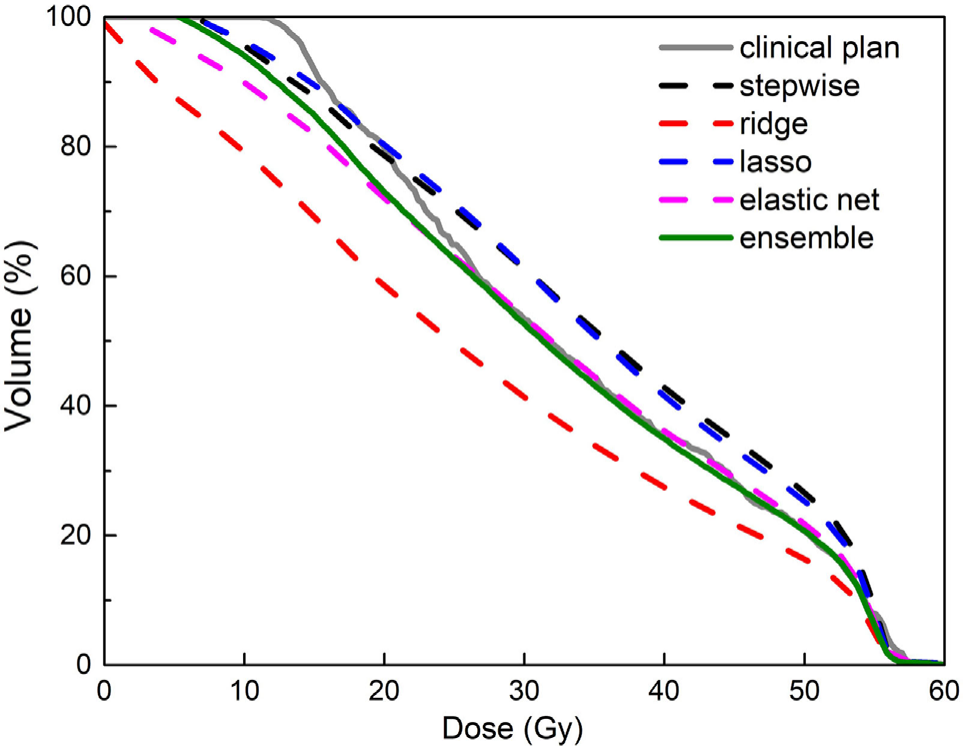
An example of improved accuracy of the ensemble model (green solid line) in predicting a bladder dose volume histogram (DVH) for a prostate plan over individual models (dashed lines in other colors). All models were trained with data that include dosimetric outliers (see Robustness to Dosimetric Outliers). The clinical plan DVH (gray solid line) is the “ground truth.” Note that the green line follows the gray line most closely.

Figure 8 shows an example of improved prediction accuracy of the proposed method, compared with other individual models. In this case, stepwise and ridge perform poorly while lasso and elastic net perform reasonably well, and the ensemble model outperforms all individual models. Note that in different situations, different models perform well, and the proposed model performs most consistently. Improved DVH prediction accuracy usually results in better plan optimization guidance (i.e., optimization constraint generation), since it provides the treatment planning system correct information of the best achievable OAR sparing without compromising PTV coverage.

Building models for different treatment sites may face different challenges. For example, the number of cases required to train a model may be different. The more complex head and neck cases require more training cases to well represent the case population, while prostate cases have fewer OARs and are generally easier to train. Second, different treatment strategies are often used to treat different sites. For example, some sites require multiple PTVs while other sites require hard constraints. Last but not least, the amount of intrinsic variance in head and neck cases are more than that of prostate cases due to potential trade-off considerations. As a result, dataset characteristics vary from treatment site to treatment site and individual model performances vary correspondingly. The ensemble model ensures the best performing model gets the highest weighting. All in all, each treatment site should be treated differently in KBP to get the best possible prediction accuracy, and the ensemble model helps to reduce the amount of effort required in terms of model selection. Ideally, the ensemble method should be trained for each treatment site, since data characteristics change from dataset to dataset. However, if there are two datasets from two treatment sites with very similar characteristics, such as DVH variability, number of cases, then it is possible to re-use the model weight $\alpha_{k}^{\text{*}}$ directly.

The main limitation of the proposed approach is the training time. Two major components of knowledge-based modeling are feature extraction and model training. The feature extraction part of the proposed model takes on average 5 s for each case, and feature extraction is done only once. Model training takes less than 10 s for each individual model. In the proposed model, individual model training is repeated by the number of component models times the number of in-model cross validation. As a result, in our hardware setup, it takes less than 10 min to run a single regression model, and it takes 30 min to run a 20-fold cross-validated ensemble model. The prediction procedure is very simple and takes less than 1 s to calculate Therefore, once a model is calculated, it can be easily stored and applied to DVH predictions.

Possible future research topics include the optimal selection of models as well as the optimal number of models in the ensemble. In this study, we limit the number of models included in the training set to avoid overfitting. While too many models in the ensemble warrant overfitting the data, the current number of models (9) is very conservative. With the regulation of the non-negative constraint, the proposed approach could potentially see further performance improvements if more models are included in the ensemble. We expect the optimal number of models in the ensemble to be dependent of the size of the dataset. In addition, the proposed methodology can be easily expanded to more complicated non-linear models. We use linear models in the ensemble due to the limitations of training dataset size. As more cases become available, more complicated models become viable.

## Author Contributions

JZ proposed the model, conducted experiments, and wrote the first draft of the manuscript. QW oversaw the workflow of the study and contributed in the clinical aspect of the study. TX extracted and pre-processed data for the experiments in the paper. YS provided suggestions regarding the study design. F-FY provided critics in the experimental design. YG contributed advice in the statistical methods and revised the manuscript.

## Conflict of Interest Statement

The authors declare that the research was conducted in the absence of any commercial or financial relationships that could be construed as a potential conflict of interest.
